# Microtiter Screening Reveals Oxygen-Dependent Antimicrobial Activity of Natural Products Against Mastitis-Causing Bacteria

**DOI:** 10.3389/fmicb.2019.01995

**Published:** 2019-08-28

**Authors:** Scott A. Ferguson, Ayana Menorca, Essie M. Van Zuylen, Chen-Yi Cheung, Michelle A. McConnell, David Rennison, Margaret A. Brimble, Kip Bodle, Scott McDougall, Gregory M. Cook, Adam Heikal

**Affiliations:** ^1^Department of Microbiology and Immunology, University of Otago, Dunedin, New Zealand; ^2^School of Chemical Sciences, The University of Auckland, Auckland, New Zealand; ^3^Deosan Ltd., Waharoa, New Zealand; ^4^Cognosco, Anexa FVC, Morrinsville, New Zealand

**Keywords:** mastitis, oxygen-dependent, *Streptococcus uberis*, *Staphylococcus aureus*, antimicrobial, natural product inhibitors, β-lapachone

## Abstract

In this study we investigated the influence of oxygen availability on a phenotypic microtiter screen to identify new, natural product inhibitors of growth for the bovine mastitis-causing microorganisms; *Streptococcus uberis*, *Staphylococcus aureus*, and *Escherichia coli.* Mastitis is a common disease in dairy cattle worldwide and is a major cause of reduced milk yield and antibiotic usage in dairy herds. Prevention of bovine mastitis commonly relies on the application of teat disinfectants that contain either iodine or chlorhexidine. These compounds are used extensively in human clinical settings and increased tolerance to chlorhexidine has been reported in both Gram-positive and Gram-negative microorganisms. As such new, non-human use alternatives are required for the agricultural industry. Our screening was conducted under normoxic (20% oxygen) and hypoxic (<1% oxygen) conditions to mimic the conditions on teat skin and within the mammary gland respectively, against two natural compound libraries. No compounds inhibited *E. coli* under either oxygen condition. Against the Gram-positive microorganisms, 12 inhibitory compounds were identified under normoxic conditions, and 10 under hypoxic conditions. Data revealed a clear oxygen-dependency amongst compounds inhibiting growth, with only partial overlap between oxygen conditions. The oxygen-dependent inhibitory activity of a naturally occurring quinone, β-lapachone, against *S. uberis* was subsequently investigated and we demonstrated that this compound is only active under normoxic conditions with a minimum inhibitory concentration and minimum bactericidal concentration of 32 μM and kills via a reactive oxygen species-dependent mechanism as has been demonstrated in other microorganisms. These results demonstrate the importance of considering oxygen-availability in high-throughput inhibitor discovery.

## Introduction

Bovine mastitis is the most common reason for antibiotic use in the dairy industry worldwide (Mcdougall, 2014; Gomes and Henriques, 2016). Application of teat antiseptics commonly containing either chlorhexidine or iodine is highly effective in reducing transmission of bacteria hence controlling the risk of mastitis (Pankey et al., 1984; Williamson and Lacy-Hulbert, 2013). Globally, use of chlorhexidine as a teat disinfectant accounts for 10–25% of the market (Hemling, 2002) and increased tolerance to chlorhexidine has been reported in both Gram-positive (Alotaibi et al., 2017; Hardy et al., 2018) and Gram-negative hospital-associated microorganisms (Guo et al., 2015).

As antibiotic use globally continues to rise against a backdrop of spreading antimicrobial resistance, the use of medically relevant human-use antimicrobials in agriculture is coming under increasing scrutiny (O’Neill, 2014; Klein et al., 2018). Additionally, antibiotic therapy of mastitis may increase the risk of selection of resistant bacteria, and as some of the same classes of medically important antibiotics used in agriculture (e.g., β-lactams) are also used in human medicine, the emergence of resistant bacteria is concerning. Discovery of antimicrobial alternatives for animal, but not human use, so-called ‘Green Antimicrobials’ (Toutain et al., 2016), for the prevention, or treatment of bacterial infections, such as bovine mastitis, would greatly assist in reducing the global usage of medically important human-use antimicrobials.

Many bacterial species have been associated with bovine mastitis, but *Streptococcus uberis*, *Staphylococcus aureus*, and *Escherichia coli* are amongst the most common. Environmental bacteria are a major cause of disease in agriculture and are, by necessity, capable of growth and survival under a range of conditions. *Streptococcus uberis*, a Gram-positive environmental pathogen, is the main cause of clinical and subclinical bovine mastitis cases in the early dry period, and around calving in New Zealand (Williamson et al., 1995; Pankey et al., 1996; Mcdougall, 1998) and is a cause of bovine mastitis throughout the world (Hogan et al., 1989; Wilson et al., 1997; Phuektes et al., 2001). *S. uberis* colonizes cows and their environment and has been isolated from the skin, lips, tonsils, gut, genital tract, teat orifice and canal, infected udders of cows as well as in bedding and pasture from the dairy herd environment (King, 1981). These diverse locations require the bacterium to not only tolerate variations in conditions for environmental growth and persistence, but also in their disease-causing location, the mammary gland, where during infection low oxygen conditions are prevalent (Mayer et al., 1988; Lopez-Benavides et al., 2007). Thus, pathogens are subjected to changes in growth conditions depending on their location (Kromker et al., 2014) and these diverse conditions are rarely, if ever, optimal for growth. The last two decades have revealed conventional antimicrobial discovery approaches, e.g., target-based and phenotypic HTS, to be largely unsuccessful (Payne et al., 2007; Tommasi et al., 2015). Bacteria are routinely grown, and antimicrobial screening conducted, under conditions optimized for growth in the laboratory (oxygenation, temperature, pH, nutrient availability), even though conditions under which the target organisms cause disease may differ considerably from laboratory conditions and in fact, may be ‘sub-optimal’ for growth (Cooper, 2013). Variations in screening conditions have been shown to identify different inhibitors both in target-based and phenotypic screens (Miller et al., 2009; Dunn et al., 2015). Furthermore, it has recently been shown that apparently simple antimicrobial killing assays are very sensitive to variations in culture conditions and bacterial growth phase (Harms et al., 2017). Anti-infectives, therefore, must also be effective under the same diverse range of conditions to prevent or treat disease (Nathan et al., 2008). In the case of oxygenation this is particularly significant, as antimicrobial efficacy has recently been directly linked to bacterial respiration and energy production (Lobritz et al., 2015) and reduced oxygen concentrations have been demonstrated within the mammary gland of cows with mastitis (Mayer et al., 1988).

The aim of this investigation was to determine the effect of oxygen, the levels of which affect cellular energy production, on microtiter screening assays to identify new antimicrobials for agricultural usage. Our results demonstrated that there was a strong oxygen dependency on the inhibitory profiles of various antimicrobial compounds against *S. uberis*. To determine the underlying molecular basis of the oxygen dependency identified by our screening approach, we selected the compound β-lapachone which was inhibitory under normal oxygen concentrations, but not inhibitory under low oxygen concentrations, for further investigation.

## Materials and Methods

### Bacterial Strains and Growth Conditions

All growth media and reagents were purchased from Sigma-Aldrich (New Zealand) unless otherwise stated and were prepared according to the manufacturers’ specifications. The *S. uberis* strain used in this investigation was a clinical bovine mastitis isolate from the Manawatu-Wanganui region of New Zealand and was kindly provided by Dr. Olaf Bork (Mastaplex, New Zealand). Recently the genome of this isolate has been sequenced and deposited under Accession No. CP022435.1 and designated as *S. uberis* NZ01 (Taiaroa et al., 2018). *S. uberis* was routinely maintained on THBA and grown in THB at 37°C with agitation (200 rpm). *Staphylococcus aureus* BB255 (Berger-Bächi, 1983) and *E. coli* MG1655 (Bachmann, 1996) were grown under the same conditions in BHI and LB, respectively. 96-well microtiter plates were inoculated with an OD_600_ of 0.005 in a final volume of 200 μL of either THB (*S. uberis*), BHI (*S. aureus)* or LB (*E. coli*) and incubated overnight at 37°C with agitation (200 rpm). Low-oxygen growth conditions for microtiter 96-well plates were established using AnaeroPack 2.5 L containers and AnaeroGen sachets (Thermo Fisher Scientific) according to the manufacturer’s instructions. Growth was monitored via OD_600_ using a Varioskan Flash plate reader.

### Phenotypic High-Throughput Screening Assays

Mastitis-causing bacteria were screened for inhibition of growth against the NPSII (NCI, Bethesda, MD, United States) library and the commercially available NPL (Selleckchem, United States). Library compounds, dissolved in DMSO, were added at a final concentration of 10 μM (or 20 μM for *E. coli*) to 96-well polystyrene microtiter plates (Thermo Fisher Scientific, New Zealand) inoculated with either *Streptococcus uberis*, *S. aureus*, or *E. coli* (final volume 200 μL). The concentration of DMSO did not exceed 2% (vol/vol) and control wells containing 2% (vol/vol) DMSO were included in each plate. Additional control wells included: un-inoculated medium (THB, BHI, or LB), and chlorhexidine gluconate (35 μM). All controls were included in technical triplicate. Plates were incubated overnight at 37°C with agitation (200 rpm) and OD_600_ were recorded using a Thermo Scientific Varioskan Flash plate reader. Assay performance was assessed by the statistical parameters Z and Z′, which take account of both data variability and signal window (Zhang et al., 1999). Z′ is a measure of the suitability of the assay set up and takes in to account the separation between negative and positive controls, Z-factor assess the impact of the screening library on the assay (Zhang et al., 1999). Statistical analysis was conducted in GraphPad Prism 8.

### Antimicrobial Susceptibility Testing

Antimicrobial susceptibility testing was performed in biological triplicate by measuring MICs by broth microdilution in polystyrene 96-well flat-bottom microtiter plates. MICs of β-lapachone, oleanolic acid, and chlorhexidine against *S. uberis* were determined. *S. uberis* cells grown overnight were diluted to an OD_600_ of 0.05 in THB and dispensed into microtiter wells. Test compounds were added to starting wells and two-fold serial dilutions were undertaken to generate a range of inhibitor concentrations in a final volume of 200 μL. Media, compound-free (untreated), and DMSO vehicle controls were included in each microtiter plate in triplicate. After 24 h incubation at 37°C and 200 rpm, the OD_600_ of wells were read using a Varioskan Flash plate reader. The MIC was reported as the lowest concentration of the test compound for which no growth occurred. To determine the MBC inhibited cells from the MIC assay were subsequently diluted 10-fold to 1 × 10^–6^, and 20 μL of each 10-fold dilution was spotted onto THBA plates.

### Cell Killing Assays Against Exponentially Growing *S. uberis*

To investigate the kinetics of cell death induced by β-lapachone, cell killing assays were conducted with *S. uberis*, using the Miles-Misra drop-plate method to measure the viability of cells in response to β-lapachone challenge against both growing and stationary phase cells (Miles et al., 1938). To establish the cell killing kinetics of β-lapachone against growing cells, an overnight culture of stationary phase *S. uberis* cells (OD_600_ ∼ 2.0) was diluted in THB to an OD_600_ of 0.05, and grown to mid-exponential phase (OD_600_ 0.5) at 37°C, with shaking (200 rpm). Once cells had reached mid-exponential phase the culture was split into 2 mL aliquots in a 7 mL glass bijou vial, and challenged with ×1 (32 μM), ×4 (128 μM), or ×8 (256 μM) the MIC of β-lapachone. An untreated control containing 1.28% (vol/vol) DMSO, equivalent to the amount of DMSO present in the ×8 sample, was also included. Following addition of the test compound, samples were obtained at 0.5, 1, 2, 4, and 6 h post-treatment. For all treatment groups, time point samples were serially diluted 10-fold in PBS, and 20 μL of each dilution was dropped in technical triplicate onto THBA plates. Plates were incubated at 37°C for 18 h. Following incubation, colonies were counted for each technical triplicate, and the mean cfu/mL was determined from at least two independent experiments.

### Cell Killing Assays Against Stationary Phase *S. uberis*

To investigate the impact of β-lapachone on stationary phase *S. uberis* cells, an overnight culture of *S. uberis* stationary phase cells (OD_600_ ∼ 2.0) was washed twice in PBS by centrifugation at 10,000 × *g*, 25°C, for 5 min. Following washing, cells were resuspended in PBS to an OD_600_ of 0.05. 1 mL of the washed cells was used per challenge group (×1, ×4, or ×8 the MIC of β-lapachone). An untreated control containing 1.28% (vol/vol) DMSO, equivalent to the amount of DMSO present in the ×8 sample, was also included. The antioxidant L-Glutathione (50 mM) was prepared as a neutralized stock solution in distilled-H_2_O and was pre-incubated for 10-min in the cell suspensions, prior to the addition of β-lapachone. Cell killing kinetics were determined as previously described.

## Results and Discussion

Growth of *S. uberis, S. aureus*, and *E. coli* for HTS was readily achieved in microtiter 96-well plates under both normoxic and hypoxic conditions, where hypoxic conditions are defined as <1% oxygen. Microtiter cultures of *S. uberis*, *S. aureus*, and *E. coli* typically reached an OD_600_ of 0.53, 1.2, and 0.9 respectively under normoxic conditions, and 0.33, 0.3–0.5, and 0.4 respectively under hypoxic conditions. The MICs of chlorhexidine gluconate against *S. uberis* and *S. aureus* displayed only minor variations between normal- and low-oxygen conditions: Supplementary Table S1. Under low-oxygen conditions, chlorhexidine MICs were approximately two- and four-fold higher for *S. uberis* and *S. aureus*, respectively. The MIC for *E. coli* (14.3 μg/mL) was several times higher than that of the Gram-positive organisms, but was not affected by oxygen levels.

To determine the effect of oxygen availability on the antimicrobial profile of these libraries, screening was conducted in microtiter plates, in duplicate, under both normoxic and hypoxic conditions. *S. uberis, S. aureus* and *E. coli* were screened against the well-characterized, NPSII library (120 compounds) and the commercially available NPL (131 compounds). The initial screening concentration for the library compounds was 20 μM and Z′ values (derived from positive and negative control wells) were 0.6–0.9 for all organisms under both low and normal-oxygen conditions, indicating excellent assay performance. However, we observed that the NPSII library contained many highly-colored compounds which, at the initial screening concentration of 20 μM, had an undesirable effect on the sample OD_600_ absorbance signals for *S. uberis* and *S. aureus*, though not *E. coli.* This was confirmed by statistical analysis as the Z-factors (<0) indicated a low separation band due to sample and control signal variation bands touching/overlapping (Zhang et al., 1999). To address the low Z-factor observed screening Gram-positives with the NPSII, we re-screened *S. aureus* under normal-oxygen conditions, as this assay had previously returned the lowest Z-factor, with a reduced compound concentration (10 μM). The reduced screening concentration had a marked effect on the sample signal distribution such that the Z-factor was improved from <0, indicating almost no confidence in the data, to a positive value (0.1), demonstrating that this assay was now suitable for HTS. This was not observed with the NPL screened against the Gram-positive organisms, where good to excellent mean Z′ and Z-factors were returned (e.g., *S. aureus* Z′ = 0.78, Z-factor = 0.70). The decision was then taken that all remaining Gram-positive organism screens would be conducted at a library compound concentration of 10 μM.

**FIGURE 1:**
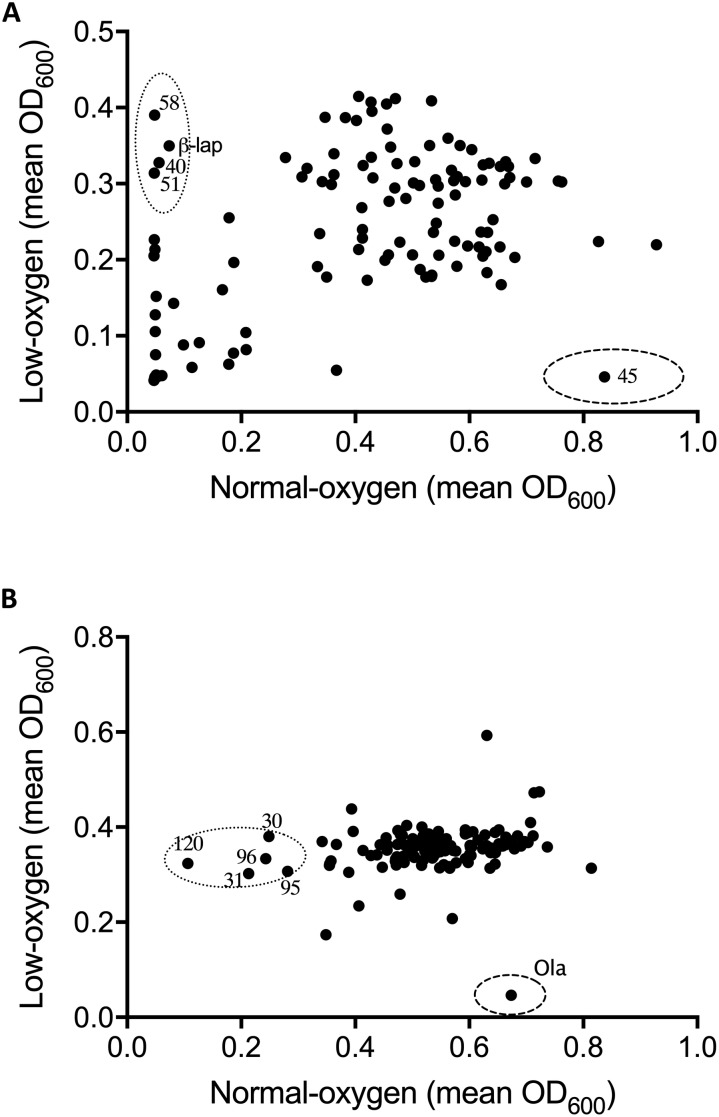
Effect of oxygen availability on the antimicrobial profile of the natural product libraries NPSII **(A)**, and NPL **(B)** against *Streptococcus uberis* NZ01. β-Lapachone (β-lap) and oleanolic acid (Ola) are indicated. Compounds that inhibited growth under normoxic conditions only are within dotted line, those inhibiting growth under hypoxic conditions are within dashed line. Results are the mean of two independent experiments.

**FIGURE 2:**
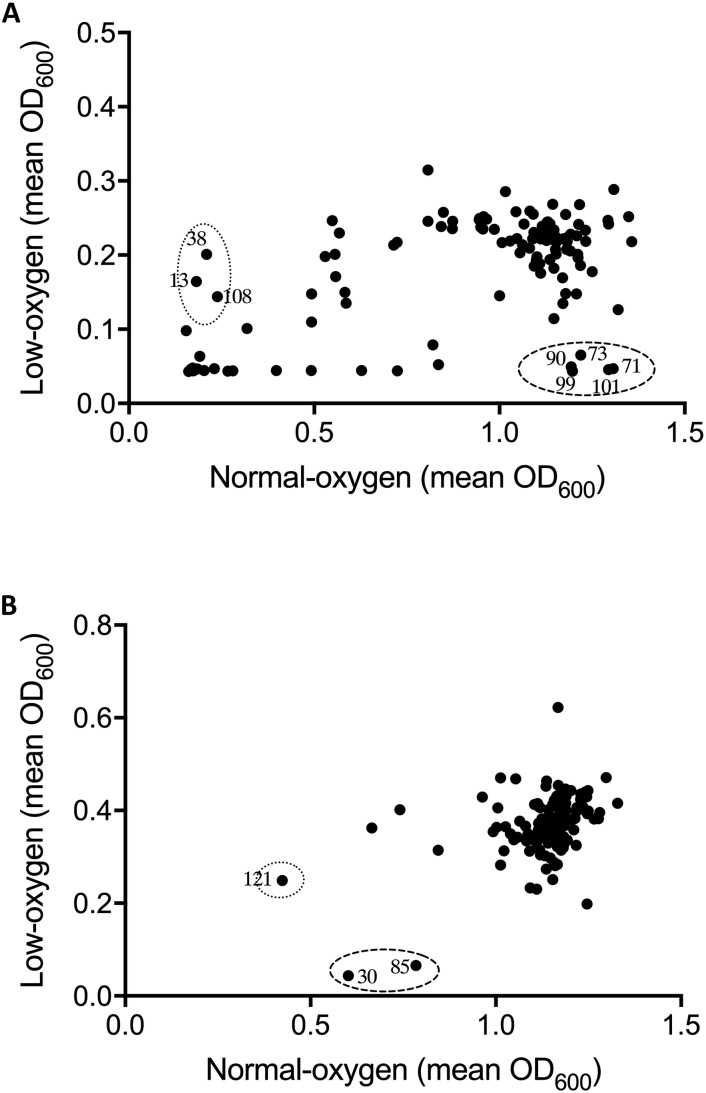
Effect of oxygen availability on the antimicrobial profile of the natural product libraries NPSII **(A)**, and NPL **(B)** against *Staphylococcus aureus* BB255. Compounds which inhibited growth under normoxic conditions only are within dotted line, those inhibiting growth under hypoxic conditions are within the dashed line. Results are the mean of two independent experiments.

Following optimization of screening conditions *S. uberis* and *S. aureus* were then re-screened, under both oxygen conditions, against NPSII and NPL at 10 μM, whereas *E. coli* was screened at a final compound library concentration of 20 μM. Results from screening assays were qualitatively assessed in terms of the inhibition of growth of mastitis causing bacteria under normoxic conditions, hypoxic conditions, or both. Neither of the libraries screened inhibited the growth of *E. coli* under either oxygen condition. However, an oxygen-dependent inhibitory effect was observed with *S. uberis* and *S. aureus* (Figures 1, 2, respectively. A number of library compounds were only capable of inhibiting growth under normoxic conditions, while others only inhibited growth under hypoxic conditions (contained within the *indicative* dotted or dashed lines Figures 1, 2, respectively). Furthermore, a collection of compounds which appeared to be capable of working under both hypoxic and normoxic conditions were identified from the NPSII, but not the NPL library against *S. uberis* (Figure 1A) and *S. aureus* (Figure 2B). Compounds which were found to be active under both oxygen conditions against *S. uberis* and *S. aureus* included the antibiotics; Antibiotic X-536A (Bovatec R), Bactobolin, and Siomycin A, as well as the toxin 4-Ipomeanol. Several other inhibitory compounds were found to be active against *S. uberis* and *S. aureus* individually, however, compounds which were inhibitory under both oxygen conditions were not examined further in this study.

Oxygen-dependent inhibition of growth was clearly observed for *S. uberis* for some compounds in the NPSII (Figure 1A) library and fewer compounds in the NPL (Figure 1B) library. Though less pronounced than for *S. uberis*, inhibition of *S. aureus* was also observed for some compounds in the NPSII library, but few compounds in the NPL library were inhibitory: Supplementary Tables S2–S5. The different compositions of these two commercially available libraries clearly contributed to the oxygen-dependency of growth inhibition. The variation observed in the compounds that inhibited growth of *S. uberis* or *S. aureus* under different oxygen conditions also suggests that the composition of libraries for antimicrobial screening should be carefully considered, based on both the final usage conditions and key target pathogens.

To further investigate the oxygen-dependency observed in our screen, we selected two inhibitory compounds (β-lapachone and oleanolic acid) that were active against *S. uberis* under differing oxygen availability. β-Lapachone is an ortho-naphthoquinone with antitumor (Silvers et al., 2017), antibacterial (Cruz et al., 1978; Pereira et al., 2006), antiparasitic (Salas et al., 2008; Dos Anjos et al., 2016), and antimalarial (De Andrade-Neto et al., 2004) properties dependent on the activity of NAD(P)H:quinone oxidoreductase (Pink et al., 2000; Bey et al., 2007). In *Bacillus subtilis* and *B. stearothermophilus* β-lapachone increases the generation of a superoxide anion ${\text{O}}_{2}^{•-}$ and hydrogen peroxide (H_2_O_2_) (Cruz et al., 1978). Based on these previous studies it is therefore likely that β-lapachone also exerts its bactericidal effects against *S. uberis* by a ROS-dependent mechanism. Consistent with this mechanism we observed that β-lapachone exerts its inhibitory and bactericidal activity against *S. uberis* growing under normoxic conditions with a MIC and MBC of 32 μM, comparable to the screening concentration used of 10 μM. To demonstrate the strict oxygen requirement for the inhibitory effect of β-lapachone, MIC assays were conducted under low-oxygen conditions. The resulting hypoxic MIC of >256 μM is in agreement with the lack of observed activity in the hypoxic screen. Further investigation of the oxygen-dependent antimicrobial activity of β-lapachone was undertaken against the beta-hemolytic mastitis-causing pathogens *Streptococcus dysgalactiae* and *Streptococcus agalactiae*. As we observed for *S. uberis*, β-lapachone inhibited *S. dysagalactiae* and *S. agalactiae* under normoxic conditions with MIC values of 32 and 64 μM respectively. However, under hypoxic conditions no (≤256 μM) β-lapachone associated antimicrobial activity was observed (Table 1). Oleanolic acid, a pentacyclic triterpenoid, inhibited *S. uberis* growth only under low-oxygen conditions, with a MIC of 128 μM, far higher than the 10 μM used in the compound library screen. The reason for this discrepancy between the measured MIC, and the concentration used in the library screen is not clear, but demonstrates the need to further validate initial results from HTS using MIC and MBC assays. Given the high MIC against *S. uberis* and that the mechanism of action of oleanolic acid against *Streptococcus mutans* UA159 (Park et al., 2017) has already been characterized, we did not investigate this compound further.

**TABLE 1:** Effect of oxygen on the minimum inhibitory, and bactericidal concentration of β-lapachone against three mastitis-causing streptococci.

	**β-Lapachone**			
	**MIC (μM)**		**MBC (μM)**	
	
	**Normoxic**	**Hypoxic**	**Normoxic**	**Hypoxic**
*Streptococcus uberis*	32	>256	32	>256
*Streptococcus dysgalactiae*	32	>256	64	>256
*Streptococcus agalactiae*	64	256	64	256

**FIGURE 3:**
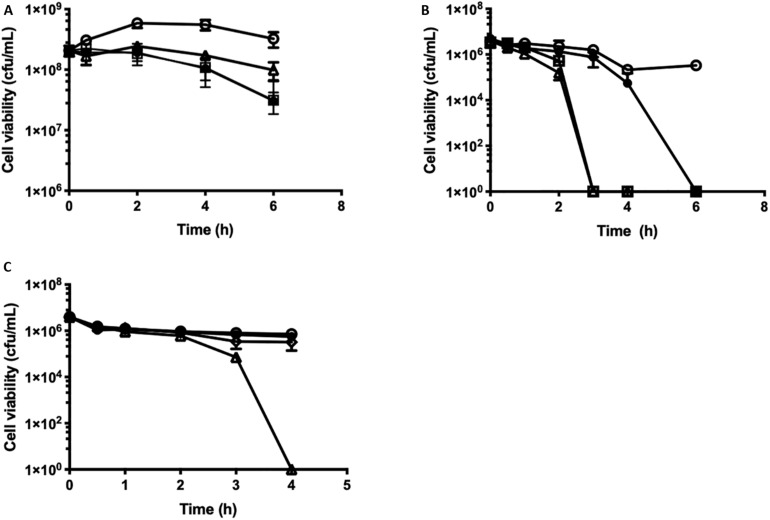
Time-dependent cell killing assays against growing and stationary phase cells of *S. uberis* in response to β-lapachone. **(A)** Growing *Streptococcus uberis* NZ01 cells at OD_600_ 0.5 (2 × 10^8^ cfu/mL) or **(B)** PBS-washed stationary phase cells at OD_600_ 0.05 (4 × 10^6^ cfu/mL) were challenged as follows with: ×1 (closed circles), ×4 (open squares), and ×8 (open triangles) MIC of β-lapachone, or untreated (open circles). **(C)** PBS-washed stationary phase *S. uberis* cells at OD_600_ 0.05 (3.93 × 10^6^ cfu/mL) were challenged as follows with: glutathione (50 mM) (closed circles), β-lapachone (×8 MIC) (open triangles), and β-lapachone (×8 MIC) with glutathione (50 mM) (open diamonds), or untreated (open circles). Error bars represent the standard deviation of the mean from three biological replicates. Where no error bars are visible, error bars are smaller than the symbol size. All cfu/mL determinations were performed in technical triplicate.

The kinetics of β-lapachone mediated cell killing were investigated by time-dependent cell viability assays against *S. uberis*. Growing cells, OD_600_ 0.5 (2 × 10^8^ cfu/mL) were challenged with increasing concentrations of β-lapachone, and the viability of the cells was determined (Figure 3A). The addition of ×1–8 MIC (32–256 μM) of β-lapachone did not induce cell lysis, as observed by the lack of change in the optical density readings between challenged and untreated cells. At ×8 MIC (256 μM), β-lapachone challenge exerted a bacteriostatic effect (Figure 3A) against exponentially growing cells with cell numbers reduced by 1-log_10_ in 6 h. No viable cells were detected 24 h post-treatment, indicating that the bactericidal activity of β-lapachone against growing cells is dependent upon duration of exposure. However, when PBS-washed stationary phase cells (4 × 10^6^ cfu/mL) were treated with β-lapachone a more pronounced bactericidal effect was observed (Figure 3B). Six hours post-treatment, ×1 MIC (32 μM) killed all viable cells, and 3 h post-treatment ×4 MIC (128 μM) killed all viable cells, indicating that stationary phase *S. uberis* cells in PBS are sensitive to the action of β-lapachone and are unable to respond to challenge by ROS. The bactericidal activity observed with β-lapachone against stationary phase cells could be prevented by the addition of the antioxidant glutathione (50 mM), confirming a ROS-dependent mechanism of killing as has been previously described for β-lapachone (Figure 3C) (Cruz et al., 1978). Protection against β-lapachone-induced ROS by glutathione has also been demonstrated in cancer cells (Park et al., 2014).

Examination of the *S. uberis* NZ01 genome (Accession No. CP022435.1), reveals that the organism has genes for detoxification of ROS, including one annotated superoxide dismutase for the conversion of ${\text{O}}_{2}^{•-}$ to H_2_O_2_ and O_2_, as well as AhpCF alkyl hydroperoxide reductase (Taiaroa et al., 2018), though the activity of these enzymes in *S. uberis* NZ01 have not yet been investigated. Analysis of the *S. uberis* NZ01 genome also reveals that to mitigate the H_2_O_2_ produced by superoxide dismutase, NZ01 encodes a gene for the flavoprotein NADH oxidase. NADH oxidase is essential in the normoxic metabolism of *S. pyogenes* (Gibson et al., 2000), and plays a key role in the oxidative stress tolerance of *S. suis* (Zheng et al., 2017).

## Conclusion

Growth and screening conditions both impact the susceptibility of microorganisms to antimicrobials (Payne et al., 2007; Tommasi et al., 2015; Harms et al., 2017). Here, we show that oxygen levels in phenotypic screening assays had a profound effect on the ability of natural products to inhibit the growth of two mastitis-causing organisms *S. uberis* and *S. aureus.* β-Lapachone was identified as an oxygen-dependent inhibitor of *S. uberis*, which exerted a time-dependent bactericidal effect, most likely mediated by accumulation of ROS. This work demonstrates that in the search for new antimicrobials exclusively for use in agriculture and with no human application, researchers should carefully consider both screening and end-usage conditions.

## Data Availability

The datasets generated for this study are available on request to the corresponding author.

## Author Contributions

SF, GC, AH, and KB conceived and designed the research. GC and AH supervised the project. SF, AM, EVZ, and C-YC performed the research. SF, DR, MB, SM, GC, and AH analyzed the data. SF and AH wrote the manuscript. All authors contributed to the manuscript revision, read, and approved the submitted version.

## Conflict of Interest Statement

KB is CEO of Deosan Ltd., manufacturers of TeatX, a chlorhexidine-based teat disinfectant. This work was, in part, funded by Deosan Ltd. The remaining authors declare that the research was conducted in the absence of any commercial or financial relationships that could be construed as a potential conflict of interest.

## Abbreviations

BHI: brain heart infusion
DMSO: dimethyl sulfoxide
HTS: high-throughput screening
LB: Luria-Bertani
MBC: minimum bactericidal concentration
MIC: minimum inhibitory concentration
NPL: Natural Product Library
NPSII: Natural Products Set II
OD_600_: optical density 600 nm
PBS: phosphate buffered saline
ROS: reactive oxygen species
THBA: Todd-Hewitt agar
THB: Todd-Hewitt Broth
vol/vol: volume/volume
