# Supplementary material for: Microtiter Screening Reveals Oxygen-Dependent Antimicrobial Activity of Natural Products Against Mastitis-Causing Bacteria
Source: Front Microbiol. 2019 Aug 28;10:1995. doi: 10.3389/fmicb.2019.01995 (PMC6722467; doi:10.3389/fmicb.2019.01995)
Supplement: Supplementary file 1 [file Data_Sheet_1.docx]

**Supplementary Information**

**Table S1.** Effect of oxygen on the minimum inhibitory concentration of chlorhexidine against the three-major mastitis-causing microorganisms^1^

|  | Chlorhexidine  MIC (μg/mL) | |
| --- | --- | --- |
|  | Normoxic | Hypoxic^2^ |
| *Streptococcus uberis* | 1.0 | 2.0 |
| *Staphylococcus aureus* | 0.45 | 1.8 |
| *Escherichia coli* | 14.3 | 14.3 |

^1^Cells were grown overnight and tested in the following media: *S. uberis* NZ01, THB; 
*S. aureus* BB255, BHI; and *E. coli* MG1655, LB. Starting OD_600_ for determining MIC’s was 0.005. Results are the mean of two independent experiments.
^2^Hypoxic conditions were generated using Oxoid AnaeroGen 2.5L Sachets.

**Table S2.** Oxygen-dependent (normoxic or hypoxic) antimicrobials from the NPSII library against *Streptococcus uberis* NZ01

| Sample ID | Compound | Structure | Oxygen |
| --- | --- | --- | --- |
|  |  |  |  |
| 20  (β-lap) | β-lapachone  CAS 4707-32-8 | 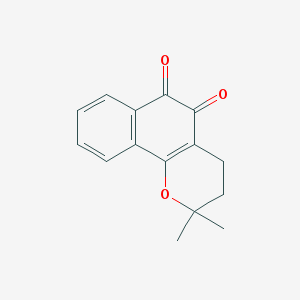 | Normoxic |
| 40 | Ellipticine  CAS 519-23-3 | 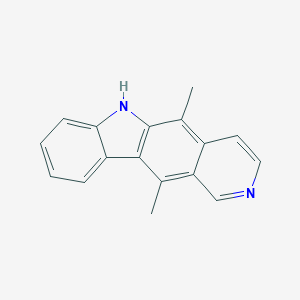 | Normoxic |
| 45 | Parthenin  CAS 508-59-8 | 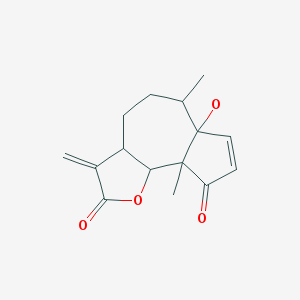 | Hypoxic |
| 51 | Tirandamycin  CAS 34429-70-4 | 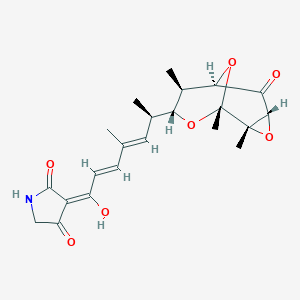 | Normoxic |
| 58 | Lankacidin C  CAS 23623-31-6 | 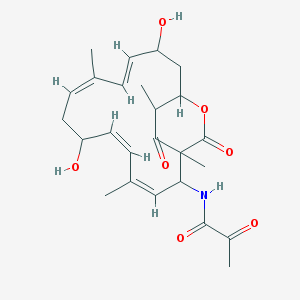 | Normoxic |

Chemical structures have been reproduced from PubChem (Kim et al., 2016)

**Table S3.** Oxygen-dependent (normoxic or hypoxic) antimicrobials from the NPL library against *Streptococcus uberis* NZ01

| Sample ID | Compound | Structure | Oxygen |
| --- | --- | --- | --- |
|  |  |  |  |
| 30 | Emodin  CAS 518-82-1 | 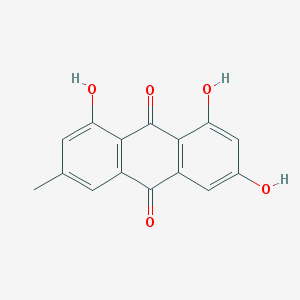 | Normoxic |
| 31 | Enoxolone  CAS 471-53-4 | 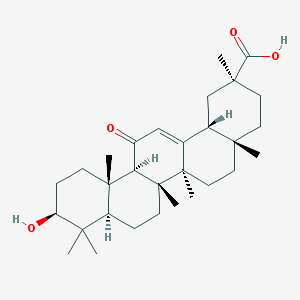 | Normoxic |
| 61  (Ola) | Oleanolic Acid  CAS 508-02-1 | 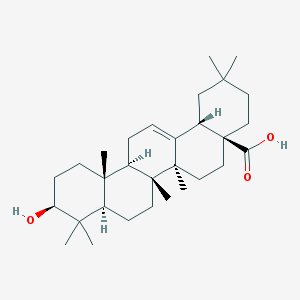 | Hypoxic |
| 95 | Ammonium Glycyrrhizinate  CAS 53956-04-0 | 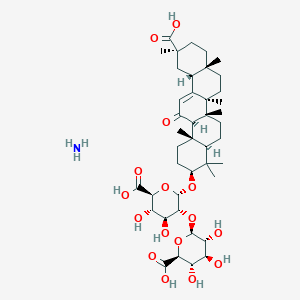 | Normoxic |
| 96 | Biochanin A  CAS 491-80-5 | 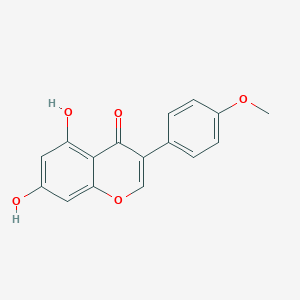 | Normoxic |
| 120 | Chrysophanic Acid  CAS 481-74-3 | 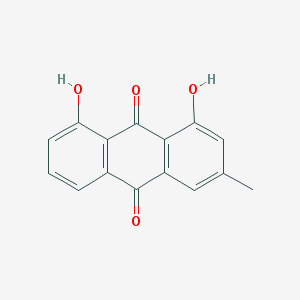 | Normoxic |

Chemical structures have been reproduced from PubChem (Kim et al., 2016)

**Table S4.** Oxygen-dependent (normoxic or hypoxic) antimicrobials from the NPSII library against *Staphylococcus aureus* BB255

| Sample ID | Compound | Structure | Oxygen |
| --- | --- | --- | --- |
|  |  |  |  |
| 13 | Aureomycin  CAS 64-72-2 | 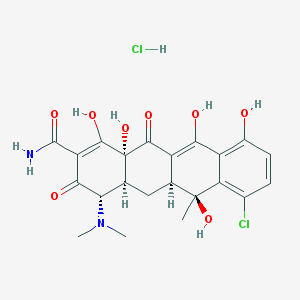 | Normoxic |
| 38 | Isopropylidine  azastreptonigrin CAS 15964-31-5 | 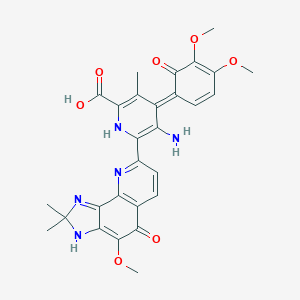 | Hypoxic |
| 71 | Gangetin  CAS 32986-79-1 | 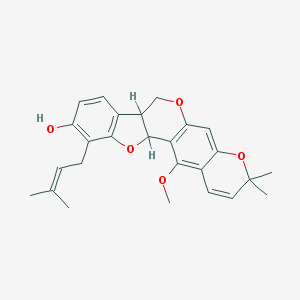 | Hypoxic |
| 73 | EHNA Hydrochloride  CAS 58337-38-5 | 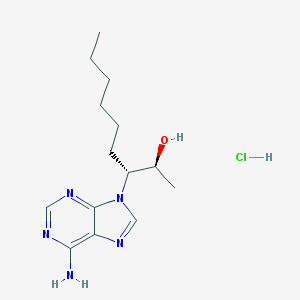 | Hypoxic |
|  |  |  |  |
|  |  |  |  |
| 90 | Chaetochromin  CAS 75514-37-3 | 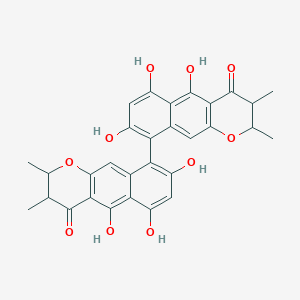 | Hypoxic |
| 99 | Dihydroergocristine  CAS 17479-19-5 | 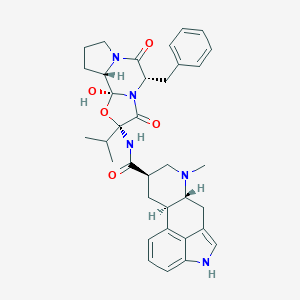 | Hypoxic |
| 101 | Michellamine B  CAS 137893-48-2 | 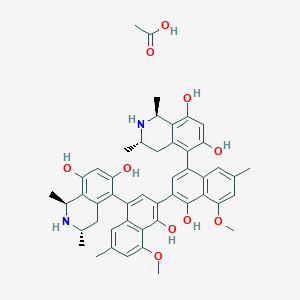 | Hypoxic |
| 108 | Toyocamycin  CAS 606-58-6 | 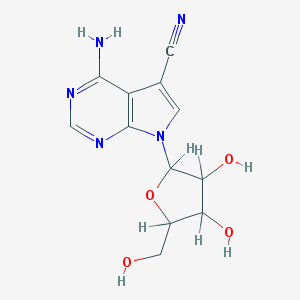 | Normoxic |
|  |  |  |  |
| Chemical structures have been reproduced from PubChem (Kim et al., 2016) | | | |

| **Table S5.** Oxygen-dependent (normoxic or hypoxic) antimicrobials from the NPL library against *Staphylococcus aureus* BB255 | | | |
| --- | --- | --- | --- |
| Sample ID | Compound | Structure | Oxygen |
|  |  |  |  |
| 30 | Emodin  CAS 518-82-1 | 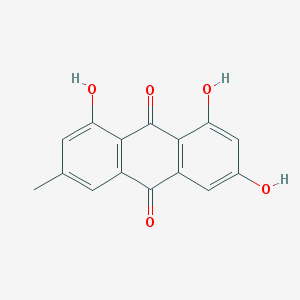 | Hypoxic |
| 85 | Tanshinone IIA  CAS 568-72-9 | 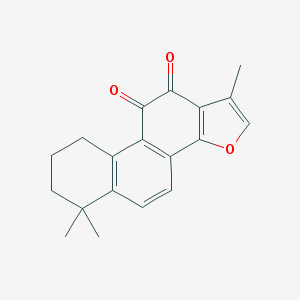 | Hypoxic |
| 121 | Curcumol  CAS 4871-97-0 | 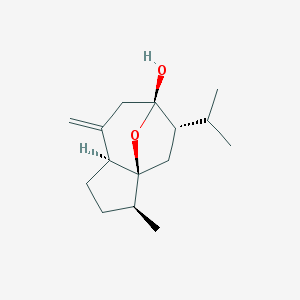 | Normoxic |

Chemical structures have been reproduced from PubChem (Kim et al., 2016)

Supplementary Reference:

Kim, S., P. A. Thiessen, E. E. Bolton, J. Chen, G. Fu, A. Gindulyte, L. Han, J. He, S. He, B. A. Shoemaker, J. Wang, B. Yu, J. Zhang, and S. H. Bryant. 2016. PubChem Substance and Compound databases. Nucleic Acids Res 44(D1):D1202-1213.
